# An Association Rule Mining Approach to Discover lncRNAs Expression Patterns in Cancer Datasets

**DOI:** 10.1155/2015/146250

**Published:** 2015-07-27

**Authors:** Paolo Cremaschi, Roberta Carriero, Stefania Astrologo, Caterina Colì, Antonella Lisa, Silvia Parolo, Silvia Bione

**Affiliations:** Computational Biology Unit, Institute of Molecular Genetics, National Research Council, Via Abbiategrasso 207, 27100 Pavia, Italy

## Abstract

In the past few years, the role of long noncoding RNAs (lncRNAs) in tumor development and progression has been disclosed although their mechanisms of action remain to be elucidated. An important contribution to the comprehension of lncRNAs biology in cancer could be obtained through the integrated analysis of multiple expression datasets. However, the growing availability of public datasets requires new data mining techniques to integrate and describe relationship among data. In this perspective, we explored the powerness of the Association Rule Mining (ARM) approach in gene expression data analysis. By the ARM method, we performed a meta-analysis of cancer-related microarray data which allowed us to identify and characterize a set of ten lncRNAs simultaneously altered in different brain tumor datasets. The expression profiles of the ten lncRNAs appeared to be sufficient to distinguish between cancer and normal tissues. A further characterization of this lncRNAs signature through a comodulation expression analysis suggested that biological processes specific of the nervous system could be compromised.

## 1. Introduction

Cancer is a highly complex disorder characterized by the dysregulation of the expression of several genes preserving cellular identity and differentiation. A comprehensive analysis of gene expression profiles in different cancer types has been performed and numerous expression signatures have been identified [1–4]. In most cases the genes described for their involvement in cancer were protein-coding oncogenes and tumor suppressors. However, in the past few years it has become increasingly clear that the human genome is pervasively transcribed and thousands of genes producing noncoding RNAs (ncRNAs) with regulatory functions were identified [5]. In particular, long noncoding RNAs (lncRNAs), transcripts longer than 200 nucleotides with no significant open reading frames, have been shown as important regulators of transcriptional and posttranscriptional events [6, 7]. This finding has prompted the researchers to investigate their role in cancer [8, 9] and several lncRNAs have been implicated in both cancer development and progression, highlighting the high genetic complexity of the disease [10].

The lncRNAs exert their functional role in cancer through various biological mechanisms and in different stages of the tumorigenic process [11]. For example HOTAIR, one of the most well-known lncRNAs, was reported as a predictor of breast cancer metastasis and poor prognosis. HOTAIR interacts with chromatin-remodeling complexes to induce heterochromatin formation in different genomic loci thus silencing gene expression [12, 13]. lncRNAs have been described also for their direct interaction with negative regulators of transcription, like in the case of lincRNA-p21 that is activated by p53 upon DNA damage and plays its role associating with hnRNP-K which acts as a transcriptional repressor [14]. However, besides these and few other examples, the lncRNAs functional mechanisms are poorly understood and their role in cancer biology remains to be fully elucidated.

An important contribution to the comprehension of lncRNAs biology in cancer could be obtained through the integrated analysis of multiple expression datasets. Traditionally, the methods used to analyze gene expression data are mostly based on the application of clustering algorithms to datasets of specific biological conditions, an approach which leads to the identification of comodulated groups of genes. However, with the growing availability of publicly available datasets, the use of new data mining techniques to integrate and to describe relationships among different types of data is highly desirable. In this perspective, the Association Rule Mining (ARM) based approaches, looking for frequent patterns in the data, have been proposed as an alternative methodology to analyze expression data [15, 16]. While this technique is commonly used in many research fields, its application in the analysis of gene expression is still limited due to the difficulties to deal with the high level of complexity and interconnection of biological processes despite several customization being proposed to overcome this issue [17–20].

In this paper, we proposed a new implementation of the ARM method for the meta-analysis of gene expression data and, in particular, to study differential expression profile of lncRNAs in multiple tumor types. The application of the ARM algorithm led us to define a total of 102 nonredundant frequent rules in lncRNAs transcriptional levels distinguishing tumor from corresponding normal tissues. We focused on the rule including the highest number of lncRNAs in brain cancers that was confirmed by independent microarray and RNA-seq datasets. Moreover, a comodulation analysis of the lncRNAs rule allowed us to shed light on putative biological processes impaired in brain tumors.

## 2. Materials and Methods

### 2.1. Long Noncoding RNA Definition

For the purpose of this study, we employed the list of lncRNAs compiled from Gencode (release 19) [21]. The selected genes corresponded to the following transcript types: 3prime_overlapping_ncrna (21), antisense (5276), lincRNA (7114), processed_transcript (515), sense_intronic (742), and sense_overlapping (202) for a total of 13870 transcripts.

### 2.2. Expression Datasets Description

For the purpose of the ARM analysis (see Section 2.3), items were represented by differentially expressed genes. Differentially expressed genes from cancer-related datasets were obtained from the CorrelaGenes database [27]. In brief, human-specific datasets were selected from the Gene Expression Omnibus (GEO) [28] Curated DataSets (GDS) and downloaded with the R package GEOquery (ver. 2.32.0) [29]. The datasets were analyzed with R package limma (ver. 3.11.1) [30]. All the results were stored in a PostgreSQL database (http://www.postgresql.org/). For this study we selected those datasets performed on the platform “Affymetrix Human Genome U133 Plus 2.0 Array” and related to cancer tissues. This selection allowed the identification of 26 datasets including 50 comparisons. From each comparison, we selected gene symbols with at least one mapped probe having an absolute value of LFC greater or equal to 1, False Discovery Rate (FDR) corrected *p* value lower than 0.05 and corresponding to a known lncRNA. This selection allowed the identification of 34 gene lists that were organized in the form of transactions for the application of the Association Rule Mining algorithm.

The ARM analysis results were compared to differentially expressed lncRNAs obtained in an independent dataset including samples from the tissues of interest. To this aim we selected the dataset E-GEOD-16011 (GSE16011) that was not present in the CorrelaGenes database. The expression set was downloaded from the ArrayExpress repository in the form of R expression set (http://www.ebi.ac.uk/arrayexpress/files/E-GEOD-16011/E-GEOD-16011.eSet.r). The expression sets were renormalized with Robust Multiarray Average (RMA) expression measure process (R package affy ver. 1.44.0) [31] and analyzed with R package limma (ver. 3.11.1) using gene annotations from platform “Affymetrix Human Genome U133 Plus 2.0 Array.”

### 2.3. Association Rule Mining Methodology

The identification of frequent patterns was performed using the Association Rule Mining algorithm implemented in the R package arules ver. 1.1.5 [32]. In the ARM formalism, datasets are organized in the form of transactions. Each transaction contains a list of elements, called items, whose nature depends on the application. In our context, each transaction corresponds to a comparison and includes all lncRNAs with at least one differentially expressed probe (absolute value ≥ 1 and FDR adjusted *p* value ≤ 0.05). The application uses the transactions to identify association rules (ARs) of the form IF A then C (A=>C). In our context, these rules can be interpreted as follows: if Set of Genes 1 is differentially expressed in a comparison then Set of Genes 2 is differentially expressed as well [16].

To measure the quality of the associations, we herein used two indexes: support and confidence. Considering two generic gene sets *X* and *Y* the two measures are defined as follows. (i) Support: the probability to find all the genes in sets *X* and *Y* differentially expressed in the same comparison. Formally Sup. = *Pr*⁡(*X* ∪ *Y*). (ii) Confidence: the probability to find all the genes in set *Y* differentially expressed in a comparison where all the genes in set *X* are differentially expressed. Formally Conf. = *Pr*⁡(*X*∣*Y*).

In our study we defined as redundant a set of rules characterized by the same set of genes or a subset of it and with the same support. In order to remove redundancy for each set of redundant rules we retained only the set including the highest number of genes (*X* ∪ *Y*).

### 2.4. Principal Component Analyses

PCA is a technique that uses an orthogonal transformation to convert a dataset onto a linear space spanned by a number of linearly independent components, named principal components, ordered by decreasing variance. The projection of the observations onto the first few principal components (i.e., PC1 and PC2) allows a reduced dimensionality maximizing the variance retained. PCA was performed with the R package FactoMineR ver. 1.29 [33]. The expression data table (Row: probes; Columns: samples) related to the DataSets GDS1962 and E-GEOD-16011 were extracted from the eSet R object and used for the PCA. In the analysis we used as variables the log2 normalized intensity values of platform probes without scaling. The different samples were used as individuals and they were labeled according to their histological classification.

### 2.5. RNA-Seq Data Analysis

RNA-seq data were used as an independent approach to validate differential expression of lncRNAs. RNA-seq data used in this study were downloaded from ArrayExpress (https://www.ebi.ac.uk/arrayexpress/) and NCBI SRA (http://www.ncbi.nlm.nih.gov/sra/) repositories. Three samples of normal brain, under the accession number E-MTAB-1733, were downloaded from ArrayExpress (ERR315477, ERR315455, and ERR315432). All tumor samples were downloaded from NCBI SRA (study SRP027383). We used three samples of glioblastoma (SRR934934, SRR934966, and SRR934911), three samples of oligodendroglioma (SRR934990, SRR934971, and SRR934734), and three samples of astrocytoma (SRR934772, SRR934784, and SRR934794). All samples share common sequencing features: they were sequenced using the Illumina HiSeq 2000 platform and a paired-end protocol (2 × 101 bp) for a total of about 60 million reads each.

Processing of RNA-seq data was performed following the protocol described in Trapnell et al. [34]. In brief, raw sra files were transformed into fastq files using SRA Toolkit available at NCBI. Raw reads were subjected to standard quality control procedures with the NGSQC-toolkit software and aligned to the human genome reference sequence (NCBI37/hg19) by the TopHat alignment software. Genes were annotated using the lncRNAs annotation file coming from Gencode (release 19). lncRNAs genes were quantified according to the TopHat-Cufflinks protocol and differential gene expression analysis was performed by CuffDiff [34]. Visualization of genomic alignments of RNA-seq reads was obtained with the IGV tool [35].

### 2.6. Comodulation Expression Analysis

The comodulation expression analysis was performed with the CorrelaGenes web application [27]. The tool uses an implementation of the Association Rule Mining algorithm based on three main customizations: (i) it extracts association rules based on two genes; (ii) one of the involved genes is constrained to be the gene selected by the user (target gene); (iii) the association indexes are calculated based on the transitions where both the target and the associated genes were present to account for the heterogeneity of the different platforms. These customizations allow CorrelaGenes to identify sets of genes whose expression appeared altered in different experimental conditions simultaneously with the target gene thus suggesting their coordinated action in the same biological process. The analysis in CorrelaGenes [27] was performed with the default parameters with the exception of copresence ≥ 10, LIFT ≥ 0, *χ* ^2^ *p* value ≤ 1. The gene Target Sign parameter was selected, for each analysis, equal to the LFC sign of the gene in brain cancer tissues (Sign +1 for ncRNA upregulated in brain cancer; Sign −1 for ncRNA downregulated in brain cancer). To improve the significance of the results we further ranked the CorrelaGenes output based on the Correlation index [36] calculated using the standard CorrelaGenes output. Only genes with a Correlation index greater than 0.3 were retained for the next step of the analysis.

### 2.7. Gene Ontology Term Enrichment Analysis and Network Visualization

The analysis of the Gene Ontology (GO) term enrichment was performed by the GOFunction R package ver. 1.14.0 [37]. The R packages biomaRt 2.20 was used to convert gene symbols into Entrez Gene IDs required by the GOFunction R package. The GO terms definition was obtained by the org.Hs.eg.db 3.0.0 R package [38]. The Benjamini correction was applied to Fisher Exact Test *p* values of enriched GO terms and considered as significant if lower than 0.05. In order to minimize the Gene Ontology (GO) term overrepresentation we selected the most specific term of each ontology (i.e., marked as “Final” in the GOFunction R package). The lists of genes associated with specific GO terms were downloaded using the QuickGO web tool (http://www.ebi.ac.uk/QuickGO/) [39].

The GeneMANIA (http://www.genemania.org/) [40] and STRING 9.1 (http://string-db.org/) [41] web tools were used to visualize the network of interactions among genes.

## 3. Results and Discussion

### 3.1. Association Rule Mining Meta-Analysis

**Figure 1:**
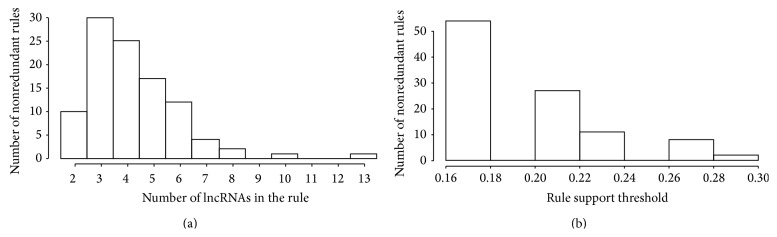
Distribution of the identified 102 nonredundant rules. (a) Distribution of identified rules based on the number of lncRNAs contained; (b) distribution of the identified rules based on support thresholds.

We applied the ARM method to identify common patterns of long noncoding RNAs differential expression distinguishing tumor samples from their respective not affected tissues. For this purpose, we selected 26 microarray datasets from the GEO Datasets Archive (http://www.ncbi.nlm.nih.gov/gds) from which a total of 34 pairwise comparisons (i.e., tumor against normal tissue) showing expression modulation for at least one lncRNA were assessed (see Section 2.2 and Supplementary Table I available online at http://dx.doi.org/10.1155/2015/146250). The lists of differentially expressed lncRNAs were used as input for the ARM algorithm. After applying a support threshold of ≥0.15, ensuring that the identified rules were present in at least 6 out of 34 comparisons tested, and a confidence threshold equal to 1, ensuring that the identified rules were confirmed in all the comparisons where the gene set is differentially expressed (i.e., the rule “if gene *X* is modulated then gene *Y* is modulated” is true in all the comparisons where the gene *X* is modulated), the ARM algorithm identified 59,542 redundant rules each including a number of lncRNAs ranging from 2 to 13. The obtained rules resulted based on the differential expression of 53 lncRNAs assorted in 102 nonredundant rules (Supplementary Tables II and III). In Figure 1 is shown the distribution of the identified 102 nonredundant rules based on (i) the number of ncRNAs contained (Figure 1(a)) and (ii) the threshold of support (Figure 1(b)).

**Figure 2:**
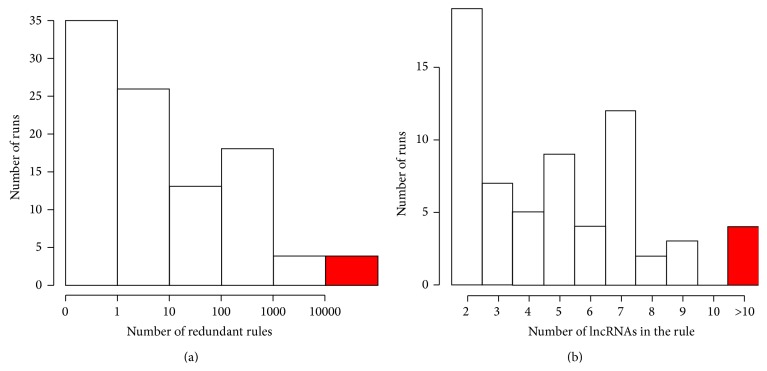
Distribution of the results of the 100 simulation runs. (a) Distribution of the number of redundant rules produced in the simulation runs; (b) distribution of the number of lncRNAs contained in the wider rule in each simulation run.

In order to verify the consistency of the results obtained we performed a simulation analysis running the ARM algorithm for 100 times on a comparable set of randomly selected comparisons and applying the same selection thresholds to extract rules. The results of the simulation test were analyzed in terms of the number of rules obtained and of the number of lncRNAs included in each rule. We found that only four simulations generated a number of redundant rules (i.e., >10.000) comparable with those found in the cancer dataset and only 4 simulations produced at least one rule containing more than 10 lncRNAs (Figure 2).

The implementation of the ARM algorithm we proposed here represents a new way to integrate heterogeneous expression data converting them in transactions that could be then compared to identify frequent patterns of differential expression. This application of the ARM method allowed us to identify 102 nonredundant rules representing frequent patterns of lncRNAs expression potentially elucidating the biological processes involved in tumorigenesis. To reduce the likelihood of generating false hypotheses, we applied a conservative confidence threshold (Conf. = 1) accounting for the limited number of comparisons available for this meta-analysis. The availability of a larger number of datasets would produce informative results even considering a lower confidence threshold. The consistence of our approach was assessed through a 100-run simulation on randomly selected datasets showing that the results obtained were unlikely due to randomness thus supporting further investigation.

### 3.2. Thirteen-Gene Rule Characterization and Validation

We concentrated our attention on the rule containing the highest number of lncRNAs (i.e., 13 lncRNAs) showing modulation of their expression in a total of six comparisons. Among the 13 lncRNAs of the rule, five (i.e., CRNDE, DLEU2, MEG3, PART1, and RFPL1S) were previously reported as involved in multiple tumor types [22, 24–26, 42] while nothing was known for six of them (i.e., KRTAP5-AS1, LINC00301, OIP5-AS1, PPP1R26-AS1, RUSC1-AS1, and UBL7-AS1). For two of the lncRNAs included in the rule (i.e., SYN2 and UHRF1), the noncoding transcript overlaps with protein-coding isoforms of the same gene thus preventing us to distinguish between the two types of molecules (Table 1).

The 13 lncRNAs rule was identified in five comparisons from the GEO dataset GDS1962 testing different kind of human brain tumors (i.e., astrocytoma grades II and III, glioblastoma grade IV, and oligodendroglioma grades II and III) against normal brain tissues. In the five comparisons, the differential expression of the 13 lncRNAs was highly consistent showing eight lncRNAs always downregulated and five lncRNAs always upregulated (Table 2). The sixth comparison supporting the 13 lncRNAs rule came from GEO dataset GDS3592 in which ovarian cancer epithelial cells were compared to normal tissue. In this comparison, the majority of the lncRNAs (10/13) resulted upregulated and seven lncRNAs (i.e., MEG3, KRTAP5-AS1, LINC00301, PART1, PPP1R26-AS1, SYN2, and CRNDE) appeared modulated in the opposite direction with respect to the brain tumor samples (Table 2).

**Figure 3:**
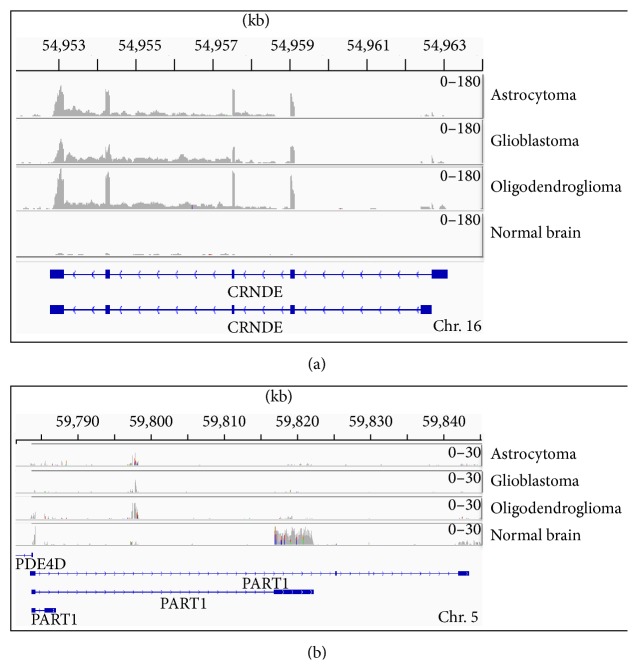
Genomic alignments of RNA-seq reads corresponding to the lncRNAs: (a) CRNDE and (b) PART1 in the three brain tumors types. The visualization of the alignment was obtained with the IGV software.

In order to assess the reliability of our findings, we exploited the E-GEOD-16011 microarray dataset downloaded from the ArrayExpress archive (https://www.ebi.ac.uk/arrayexpress/) and RNA-seq data from NCBI SRA study SRP027383 including brain tumor samples with an histological classification comparable to the ones in the GDS1962 dataset. The validation of ovarian cancer data could not be performed due to the unavailability of comparable expression datasets. From the analysis of expression profiles obtained in the E-GEOD-16011 and in the SRP027383 RNA-seq study, we were able to confirm the altered expression of six lncRNAs (i.e., RFPL1S, KRTAP5-AS1, PART1, and SYN2 which appeared consistently downregulated and DLEU2 and UHRF1 which appeared consistently upregulated). The expression of four of the 13 lncRNAs was considered as consistent with previous findings although they showed less severe modulation of their transcription levels (i.e., OIP5-AS1 and UBL7-AS1) or their expression values could not be assessed in all samples tested (i.e., CRNDE and RUSC1-AS1). Three lncRNAs were not validated: two of them (i.e., LINC00301 and PPP1R26-AS1) resulted not significantly modulated in the RNA-seq analysis and the MEG3 lncRNA appeared modulated in two out of three samples but with discordant values (Table 3). In Figure 3, the expression profiles of the CRNDE and PART1 lncRNAs from RNA-seq data were shown as example (the expression profiles of the eight remaining lncRNAs were shown in Supplementary Figure 1). Thus, we were able to confirm the altered expression of 10 out of the 13 lncRNAs identified by the ARM method on GDS1962.

Among the 10 confirmed lncRNAs, four were previously described as involved in the genesis of different tumors. In particular, CRNDE appeared to be upregulated in colorectal cancer, leukemia, and gliomas concordantly with our observations [22, 26]. DLEU2 was known to be frequently deleted in lymphocytic leukemia [24], while our study revealed an upregulation of its expression in gliomas suggesting a tissue-specific regulation of this gene. Interestingly, three out of 10 lncRNAs were previously identified as part of a signature able to distinguish among different types and grades of gliomas [26, 42]. Consistently with the signatures of Zhang et al., identified using the same datasets of the present analysis, we reported the differential expression of CRNDE, PART1, and RFPL1S. The lack of a complete overlap between the studies could be due to three main factors: (i) different criteria to select probes mapped to lncRNAs; (ii) a different statistical model for the identification of differential expressed genes, or (iii) a different study design to identify gene signatures. These observations, validated in different datasets and confirmed by previous studies, suggest that the ARM method was a suitable approach to identify set of genes whose altered expression is peculiar of brain tumor.

### 3.3. Principal Component Analysis

**Figure 4:**
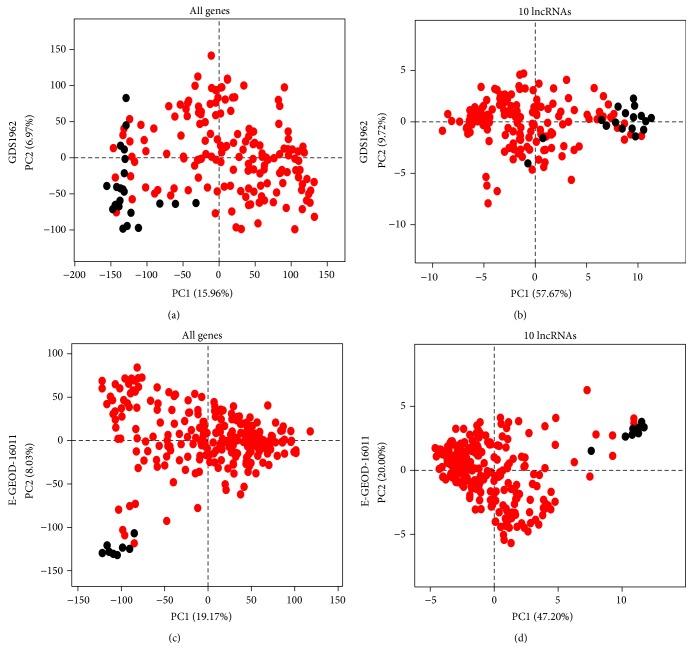
Principal Component Analysis (PCA) performed on the GEO dataset GDS1962 (a and b) and ArrayExpress dataset E-GEOD-16011 (c and d) considering intensity values of all probes (a and c) or only probes corresponding to the 10 lncRNAs (b and d). Red dots correspond to brain tumor samples and black dots correspond to normal brain samples.

In order to investigate the power of the 10 lncRNAs rule to distinguish among brain tumor and normal samples, we performed a Principal Component Analysis (PCA) using the probe intensity values from GEO dataset GDS1962 as variables.  Figure 4 showed principal components (PC) 1 and 2 obtained using intensities of all probes (Figure 4(a)) or only probes corresponding to the 10 lncRNAs (Figure 4(b)). In both analyses, the majority of normal brain samples appeared as a separate cluster distinguishable from tumor tissues. This observation was confirmed by the PCA performed on ArrayExpress dataset E-GEOD-16011 (Figures 4(c) and 4(d)) that showed similar pattern of clustering among normal and tumor samples. Moreover, a certain degree of clustering was also appreciable when tumor samples were labeled according to tumor type and grade (Supplementary Figure 2).

The PC analysis performed on the two independent datasets suggested that the 10 lncRNAs expression levels were sufficient to clearly separate samples belonging to the two groups.

### 3.4. Comodulation Gene Expression Analysis

In order to get insight into the putative involvement of the 10 long noncoding molecules in specific biological processes, we performed a comodulation analysis. For this purpose, we exploited our CorrelaGenes tool [27] looking for set of genes altered in their expression levels simultaneously with the up- or downregulation of each of the 10 lncRNAs. The CorrelaGenes tool (http://www.igm.cnr.it/cabgen/web-correlagenes0/) was queried for each lncRNAs with LFC > +1 or LFC < −1 according to their sign in the rule, in order to identify genes showing significant alteration of their expression (i.e., |LFC| > 1) in a significant proportion of comparisons tested. The analyses resulted in a total of 10 gene lists including a number of genes between 1675 and 6601 (Supplementary Tables S4 and S5). For each gene list, an enrichment analysis for Gene Ontology terms was conducted by means of the R/Bioconductor GO-function package [37] using up- or downregulated genes separately (Supplementary Tables S6 and S7).

**Figure 5:**
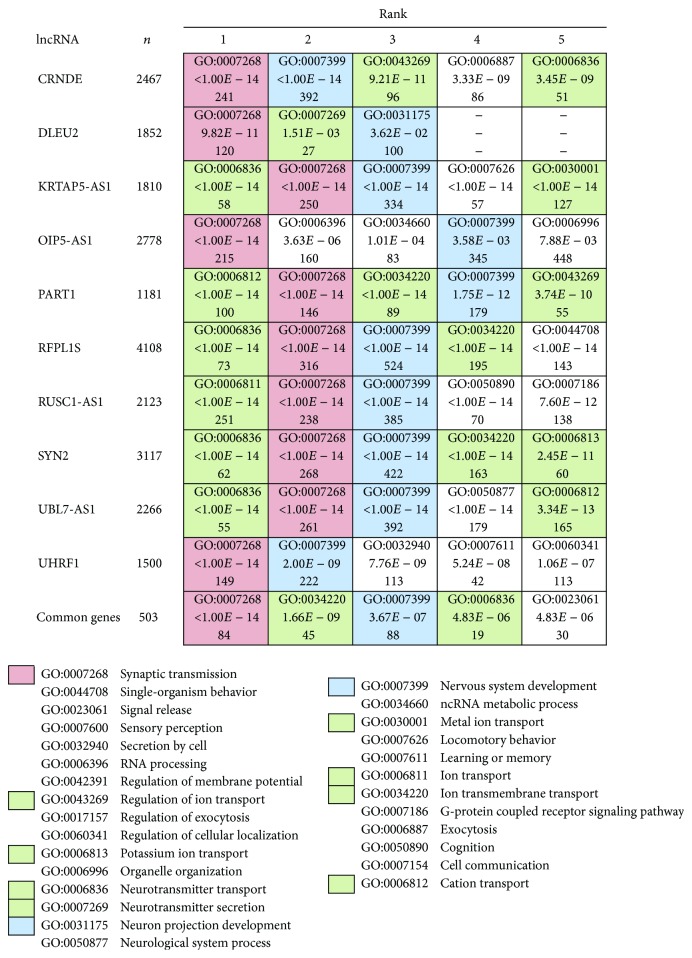
Enrichment analysis of downregulated genes from comodulation results.

For all the 10 lists of downregulated genes, the analysis showed highly significant enrichments mainly concentrated in three categories: (i) “Synaptic transmission” (GO:0007268), (ii) “Ion transport” (GO:0006811) and related terms, and (iii) “Nervous System Development” (GO:0007399). The analysis of a list of 503 “common” genes, found in at least nine out of the 10 lists, confirmed the enrichment for the same categories (Figure 5 and Supplementary Figure 3). Interestingly, these results appeared highly consistent with the neuronal enriched GO categories found in the article of Liu and coauthors [43]. In this paper, authors performed an analysis of miRNAs differential expression in pediatric gliomas together with a GO terms enrichment analysis of miRNA target genes resulting in the identification of several neuronal GO categories belonging to the “Synaptic transmission” and “Nervous System Development” clades. Any GO term related to the “Ion transport” category resulted significantly enriched in the work of Liu and colleagues leading us to speculate about a specific role of lncRNAs in this specific biological process.

Taking into consideration the upregulated transcripts, the number of “common genes” resulted highly reduced (i.e., *n* = 150) and, as expected, not significantly enriched for any GO term. However, the analysis of single gene lists allowed us to group some recurrent GO terms in three enriched categories: (i) “Cell cycle” (GO:0007049) and related terms such as “Mitotic cell cycle” (GO:0000278), “Cell cycle process” (GO:0022402), and “Cell cycle checkpoint” (GO:0000075), enriched in seven out of 10 gene lists (with adjusted *p* values ranging from 1 × 10^−14^ to 1 × 10^−2^); (ii) the “RNA metabolic process” (GO:0016070) which includes terms such as “mRNA metabolic process” (GO:0016071), “RNA splicing” (GO:0008380), and “Regulation of mRNA stability” (GO:0043488), enriched in five out of 10 gene lists (with adjusted *p* values ranging from 1 × 10^−9^ to 1 × 10^−3^); (iii) the “Gene expression” (GO:0010467) to which belong terms as “Regulation of transcription from RNA polymerase II promoter” (GO:0006357) and “Positive regulation of gene expression” (GO:0010628), enriched in four out of 10 gene lists (with adjusted *p* values ranging from 1 × 10^−6^ to 1 × 10^−3^).

**Figure 6:**
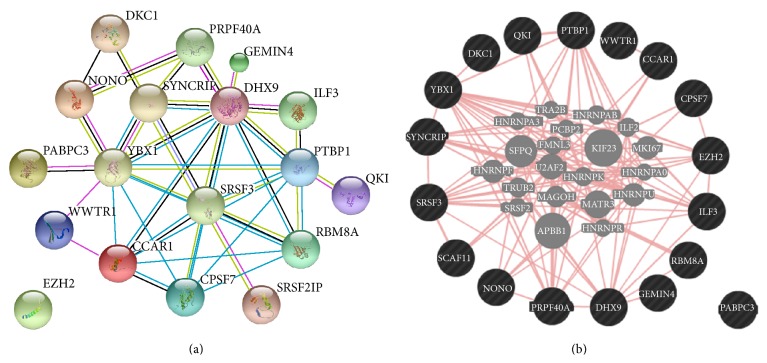
Gene networks of the selected 18 genes obtained by the tools: (a) STRING 9.1 and (b) GeneMANIA.

Among several other features, we focused on the “RNA metabolic process” category that includes many genes involved in posttranscriptional modification pathways. Taking into account all genes annotated in the “RNA metabolic process” category and all its children terms, a pool of 109 genes were found present in at least seven out of the 10 lists of upregulated genes. A functional analysis performed using both STRING and GeneMANIA tools allowed us to select a core of 18 genes highly interconnected on the basis of experiments/database or physical interactions annotations, respectively, implemented in the two tools (Figure 6).

The investigation of downregulated genes resulted highly concordant in the 10 gene lists and highlighted the putative impairment of neuronal development and functionality according to brain tumors characteristics. The analysis of the 10 lists of upregulated genes showed the enrichment of a wider range of biological processes. In agreement with the tumorigenic model, many genes showing an increase of their transcriptional levels were related to different aspects of the cell cycle. Moreover, the involvement of posttranscriptional regulation mechanisms was demonstrated by a relative enrichment of the “RNA metabolic process” GO category. A detailed characterization of upregulated genes belonging to this clade allowed us to identify a subset of 18 genes whose correlations were independently supported by different kind of studies as, for example, between YBX1 and SYNCRIP [44–46] or between CCAR1 and WWTR1 [47]. The 18 genes selected appeared to operate in several mechanisms of posttranscriptional regulation such as ILF3 in pre-mRNA splicing, mRNA cytoplasmic export, and mRNA stability [48] or QKI in alternative splicing [49]. Remarkably, some studies already demonstrated the impact of expression alterations on cell cycle and proliferation of some of these genes like SRSF3 [50] and EZH2 [51].

## 4. Conclusions

In this paper, we described the implementation of the Association Rule Mining methodology for the meta-analysis of gene expression data. The application of the ARM method resulted in the identification of a 10 lncRNAs pattern that was validated in two independent datasets of brain tumors expression data. Throughout a Principal Component Analysis, we assessed the potential of the 10 lncRNAs rule to distinguish between cancer and normal tissues. Moreover, by a comodulation analysis, we were able to outline some specific biological processes that could be putatively related to the altered expression of the 10 lncRNAs. In conclusion, we proposed this new ARM-based approach as a valuable tool to extract relevant biological information in the form of common expression patterns.

## Supplementary Material

Supplementary Material The list of comparisons from GEO datasets is provided in Table S1. The list of the 53 lncRNAs contained in at least one of the 102 nonredundant rules identified by the ARM analysis is provided in Table S2. The list of the 102 nonredundant rules identified through the ARM analysis is provided in Table S3. Number of comodulated genes for each of the 10 lncRNAs is provided in Table S4. Results of the CorrelaGenes analyses of the 10 lncRNAs are provided in Table S5. Results of the enrichment analyses of GO terms for the upregulated genes are provided in Table S6. Results of the enrichment analyses of GO terms for the downregulated genes are provided in Table S7. Figure S1. Genomic alignments of RNA-seq reads corresponding to the eight remaining lncRNAs in the three brain tumors types: (A) UHRF1, (B) UBL7-AS1, (C) DLEU2, (D) SYN2, (E) RFPL1S, (F) KRTAP5-AS1, (G) OIP5-AS1 and (H) RUSC1- AS1. In each panel the transcript annotations from both RefSeq and Gencode were displayed. Figure S2. Principal Component Analysis (PCA) performed on the GEO dataset GDS1962 (panels A and B) and ArrayExpress dataset E-GEOD-16011 (panels C and D) considering intensity values of all probes (panels A and C) or only probes corresponding to the 10 lncRNAs (panel B and D). Figure S3. Direct acyclic graph of the brain-specific Gene Ontology terms obtained by the analysis of downregulated genes.

## Figures and Tables

**Table 1:** List of the 13 lncRNAs.

Number	lncRNA symbol	lncRNA name	Reference
1	CRNDE	Colorectal neoplasia differentially expressed	Ellis et al., 2012 [22] Zhang et al., 2012 [23]
2	DLEU2	Deleted in lymphocytic leukemia 2	Lerner et al., 2009 [24]
3	KRTAP5-AS1	KRTAP5-1/KRTAP5-2 antisense RNA 1	
4	LINC00301	Long intergenic non-protein coding RNA 301	
5	MEG3	Maternally expressed 3	Wang et al., 2012 [25] Zhang et al., 2012 [23]
6	OIP5-AS1	OIP5 antisense RNA 1	
7	PART1	Prostate androgen-regulated transcript 1	Zhang et al., 2013 [26]
8	PPP1R26-AS1	PPP1R26 antisense RNA 1	
9	RFPL1S	RFPL1 antisense RNA 1	Zhang et al., 2012 [23]
10	RUSC1-AS1	RUSC1 antisense RNA 1	
11	SYN2^∗^	Synapsin II	
12	UBL7-AS1	UBL7 antisense RNA 1	
13	UHRF1^∗^	Ubiquitin-like with PHD and ring finger domains 1	

^∗^lncRNA not distinguishable from the protein coding isoform.

**Table 2:** LFC of the 13 lncRNAs in GEO datasets.

lncRNA symbol	Gene ID	lncRNA name	GDS1962					GDS3592
			Astrocytoma (grade II)	Astrocytoma (grade III)	Glioblastoma (grade IV)	Oligodendroglioma (grade II)	Oligodendroglioma (grade III)	Ovarian cancer epithelial cells
OIP5-AS1	729082	OIP5 antisense RNA 1	−1	−1.3	−1.5	−1	−1.5	−1.3
RFPL1S	10740	RFPL1 antisense RNA 1	−2.5	−2.6	−3.8	−2.3	−3.3	−2.5
MEG3	55384	Maternally expressed 3	−2.4	−2.8	−2.7	−2.6	−2.7	1.2
KRTAP5-AS1	338651	KRTAP5-1/KRTAP5-2 antisense RNA 1	−1.7	−1.7	−2	−1.1	−1.8	1
LINC00301	283197	Long intergenic non-protein coding RNA 301	−2.2	−1.5	−1.9	−1.4	−2.1	1.9
PART1	25859	Prostate androgen-regulated transcript 1	−1.4	−1.7	−2	−1.4	−1.9	2.4
PPP1R26-AS1	100506599	PPP1R26 antisense RNA 1	−1.4	−1.4	−1.2	−1.1	−1.4	1.9
SYN2	6854	Synapsin II	−2.6	−2.6	−4	−2.5	−3.8	2.2
CRNDE	643911	Colorectal neoplasia differentially expressed	3.2	3.6	4.2	1.8	3.7	−4.3
RUSC1-AS1	284618	RUSC1 antisense RNA 1	1.6	1.5	1.2	1.4	1.5	2
UBL7-AS1	440288	UBL7 antisense RNA 1	1.8	1.6	1.5	1.4	1.8	1.6
DLEU2	8847	Deleted in lymphocytic leukemia 2	1	1	1.5	1	1.4	1.5
UHRF1	29128	Ubiquitin-like with PHD and ring finger domains 1	2.5	3.6	4	3.1	3.8	3.4

**Table 3:** LFC of the 13 lncRNAs in different brain cancer datasets.

		OIP5-AS1	RFPL1S	MEG3	KRTAP5-AS1	LINC00301	PART1	PPP1R26-AS1	SYN2	CRNDE	RUSC1-AS1	UBL7-AS1	DLEU2	UHRF1
GDS1962	Astrocytoma (grade II)	−1.0	−2.5	−2.4	−1.7	−2.2	−1.4	−1.4	−2.6	3.2	1.6	1.8	1.0	2.5
	Astrocytoma (grade III)	−1.3	−2.6	−2.8	−1.7	−1.5	−1.7	−1.4	−2.6	3.6	1.5	1.6	1.0	3.6
	Glioblastoma (grade IV)	−1.5	−3.8	−2.7	−2.0	−1.9	−2.0	−1.2	−4.0	4.2	1.2	1.5	1.5	4.0
	Oligodendroglioma (grade II)	−1.0	−2.3	−2.6	−1.1	−1.4	−1.4	−1.1	−2.5	1.8	1.4	1.4	1.0	3.1
	Oligodendroglioma (grade III)	−1.5	−3.3	−2.7	−1.8	−2.1	−1.9	−1.4	−3.8	3.7	1.5	1.8	1.4	3.8

E-GEOD-16011	Astrocytoma (grade II)	−1.0	−3.8	−2.7	−1.0	−0.3	−2.9	−0.3	−3.8	2.8	n.s.	0.5	1.4	2.9
	Astrocytoma (grade III)	−1.5	−4.6	−3.8	−1.1	−0.3	−3.4	−0.4	−5.4	3.7	0.8	0.9	1.9	3.2
	Glioblastoma (grade IV)	−1.8	−4.8	−3.7	−1.1	−0.3	−3.3	−0.2	−5.4	4.4	n.s.	0.8	1.4	3.6
	Oligodendroglioma (grade II)	−1.0	−3.1	−2.7	−1.0	−0.3	−3.1	−0.3	−3.9	n.s.	1.0	0.7	1.6	3.7
	Oligodendroglioma (grade III)	−1.5	−3.6	−3.7	−1.0	−0.3	−3.3	−0.3	−5.1	2.8	1.1	0.8	1.8	3.4

RNAseq	Astrocytoma	−0.3	−2.2	n.s.	−3.0	n.s.	−3.0	n.s.	−2.5	4.7	1.5	2.0	2.3	2.5
	Glioblastoma	−0.3	−4.7	0.6	−3.2	n.s.	−4.2	n.s.	−2.2	4.7	n.s.	1.8	2.1	1.8
	Oligodendroglioma	−0.4	−1.7	−2.0	−1.4	n.s.	−1.0	n.s.	−3.8	4.9	1.0	2.3	4.0	2.5
