# Supplementary material for: An Association Rule Mining Approach to Discover lncRNAs Expression Patterns in Cancer Datasets
Source: Biomed Res Int. 2015 Jul 27;2015:146250. doi: 10.1155/2015/146250 (PMC4530207; doi:10.1155/2015/146250)
Supplement: Supplementary file 1 — Supplementary Material The list of comparisons from GEO datasets is provided in Table S1. The list of the 53 lncRNAs contained in at least one of the 102 nonredundant rules identified by the ARM analysis is provided in Table S2. The list of the 102 nonredundant rules identified through the ARM analysis is provided in Table S3. Number of comodulated genes for each of the 10 lncRNAs is provided in Table S4. Results of the CorrelaGenes analyses of the 10 lncRNAs are provided in Table S5. Results of the enrichment analyses of GO terms for the upregulated genes are provided in Table S6. Results of the enrichment analyses of GO terms for the downregulated genes are provided in Table S7. Figure S1. Genomic alignments of RNA-seq reads corresponding to the eight remaining lncRNAs in the three brain tumors types: (A) UHRF1, (B) UBL7-AS1, (C) DLEU2, (D) SYN2, (E) RFPL1S, (F) KRTAP5-AS1, (G) OIP5-AS1 and (H) RUSC1- AS1. In each panel the transcript annotations from both RefSeq and Gencode were displayed. Figure S2. Principal Component Analysis (PCA) performed on the GEO dataset GDS1962 (panels A and B) and ArrayExpress dataset E-GEOD-16011 (panels C and D) considering intensity values of all probes (panels A and C) or only probes corresponding to the 10 lncRNAs (panel B and D). Figure S3. Direct acyclic graph of the brain-specific Gene Ontology terms obtained by the analysis of downregulated genes. [file 146250.f1.zip › 146250.f1/Suppl_Fig1_revision2.pdf]

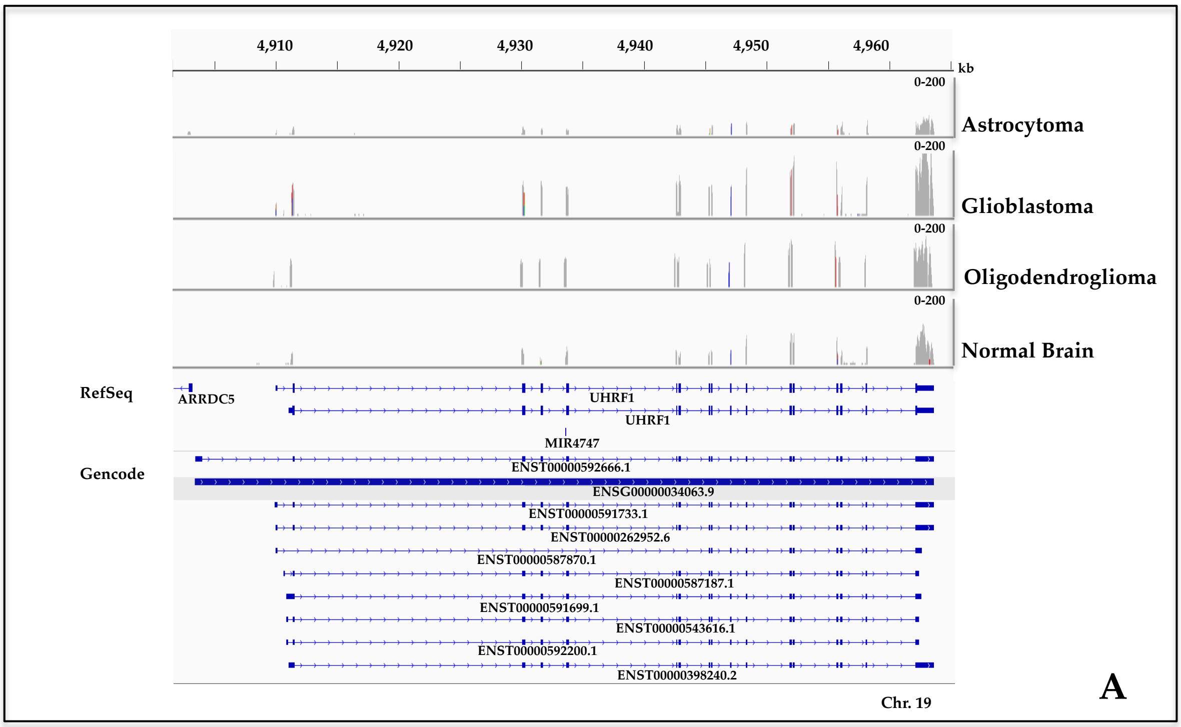
4,910
4,920
4,930
4,940
4,950
4,960
kb
0-200
Astrocytoma
Glioblastoma
Oligodendroglioma
Normal Brain
RefSeq
ARRDC5
UHRF1
UHRF1
MIR4747
Gencode
ENST00000592666.1
ENSG00000034063.9
ENST00000591733.1
ENST00000262952.6
ENST00000587870.1
ENST00000587187.1
ENST00000591699.1
ENST00000543616.1
ENST00000592200.1
ENST00000398240.2
Chr. 19
A

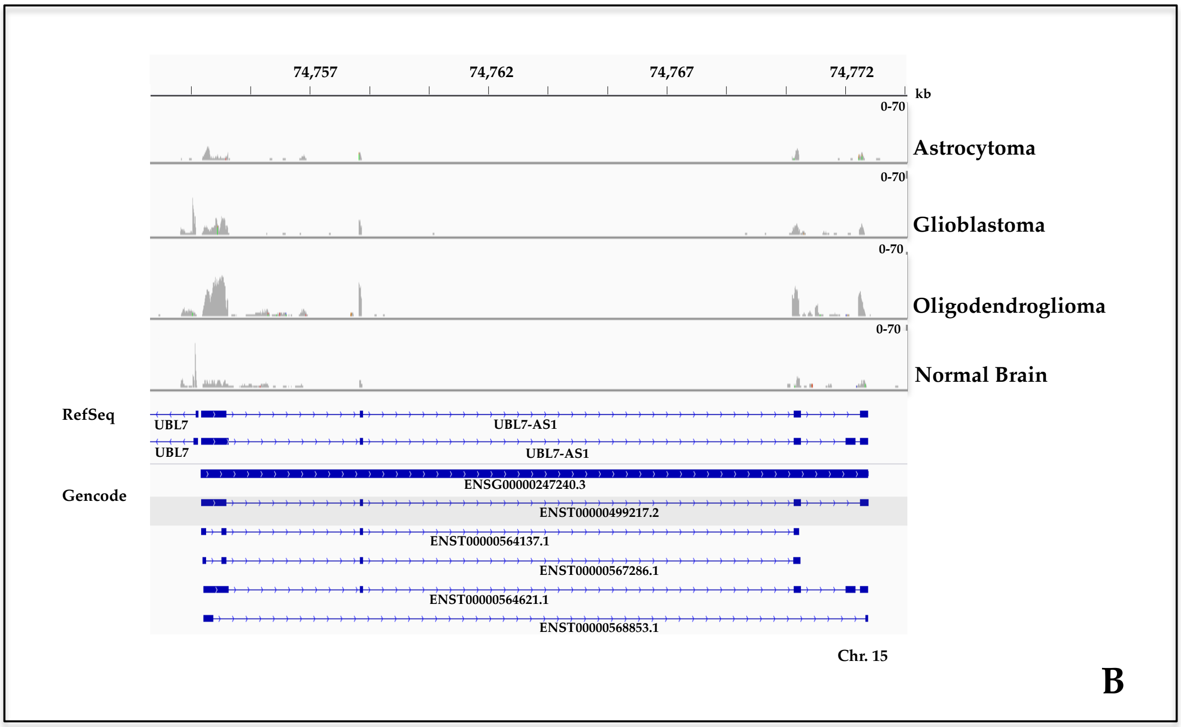
74,757
74,762
74,767
74,772
kb
0-70
Astrocytoma
Glioblastoma
Oligodendroglioma
Normal Brain
RefSeq
UBL7
UBL7-AS1
UBL7
UBL7-AS1
Gencode
ENSG00000247240.3
ENST00000499217.2
ENST00000564137.1
ENST00000567286.1
ENST00000564621.1
ENST00000568853.1
Chr. 15
B

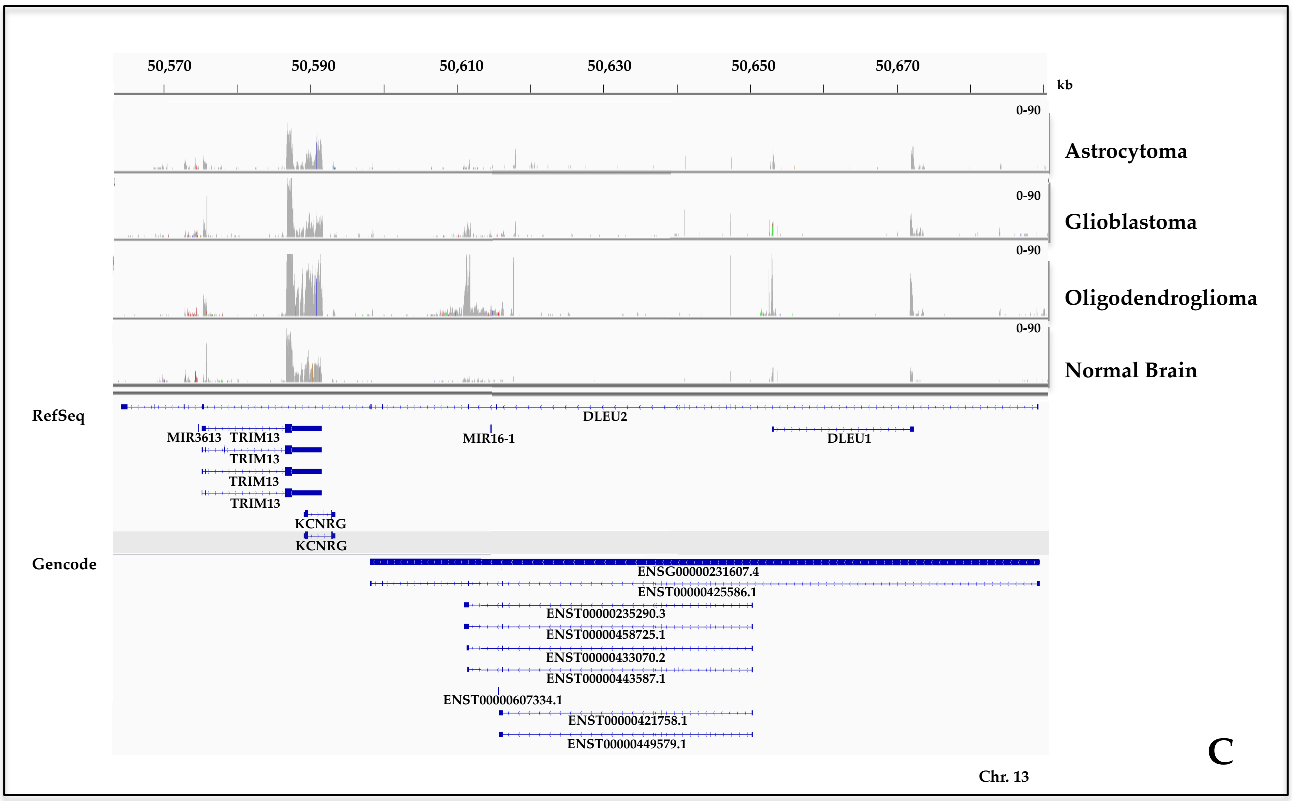

50,570
50,590
50,610
50,630
50,650
50,670
kb
0-90
Astrocytoma
0-90
Glioblastoma
0-90
Oligodendroglioma
0-90
Normal Brain
RefSeq
DLEU2
MIR3613 TRIM13
MIR16-1
DLEU1
TRIM13
TRIM13
TRIM13
KCNRG
KCNRG
Gencode
ENSG00000231607.4
ENST00000425586.1
ENST00000235290.3
ENST00000458725.1
ENST00000433070.2
ENST00000443587.1
ENST00000607334.1
ENST00000421758.1
ENST00000449579.1
Chr. 13
C

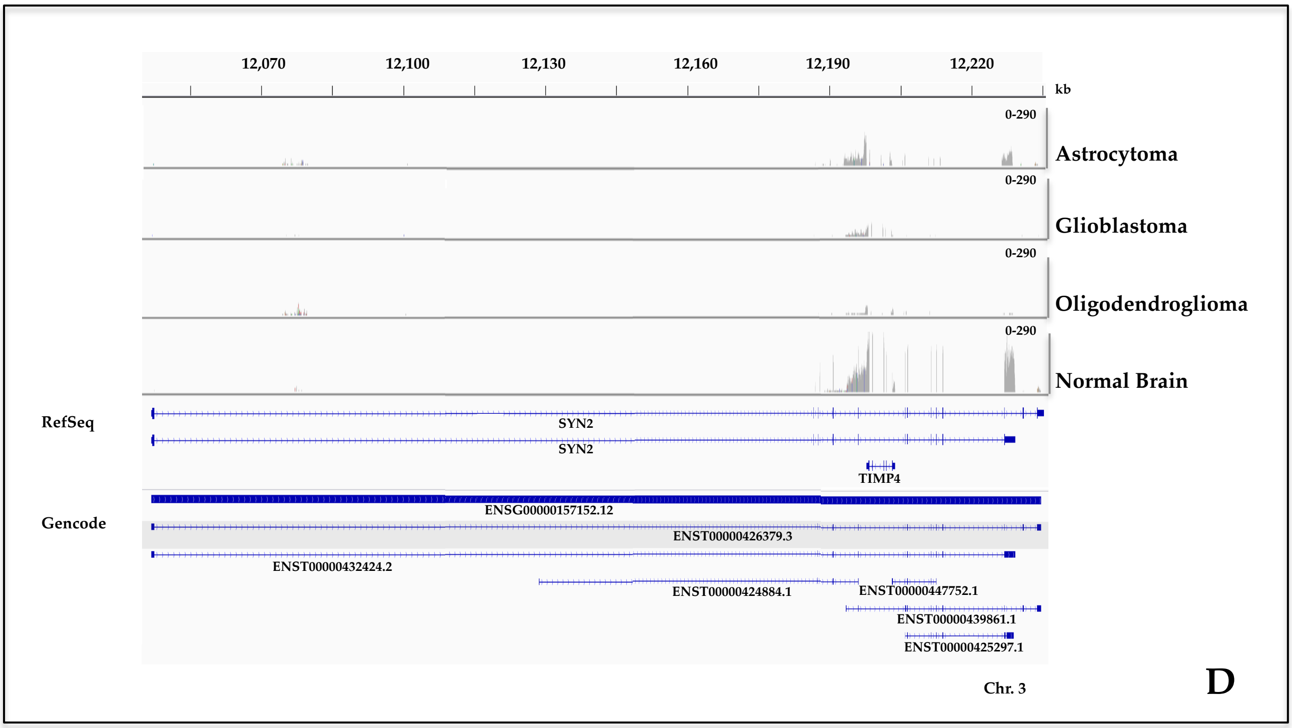

12,070
12,100
12,130
12,160
12,190
12,220
kb
0-290
Astrocytoma
0-290
Glioblastoma
0-290
Oligodendroglioma
0-290
Normal Brain
RefSeq
SYN2
SYN2
TIMP4
Gencode
ENSG00000157152.12
ENST00000426379.3
ENST00000432424.2
ENST00000424884.1
ENST00000447752.1
ENST00000439861.1
ENST00000425297.1
Chr. 3
D

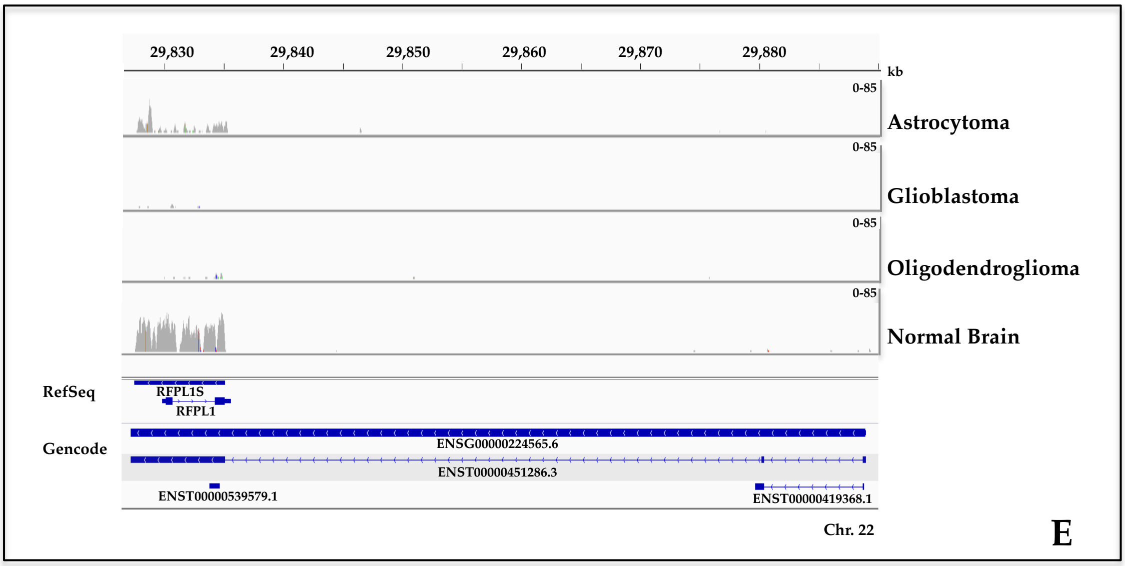
29,830
29,840
29,850
29,860
29,870
29,880
kb
0-85
Astrocytoma
0-85
Glioblastoma
0-85
Oligodendroglioma
0-85
Normal Brain
RefSeq
RFPL1S
RFPL1
Gencode
ENSG00000224565.6
ENST00000451286.3
ENST00000539579.1
ENST00000419368.1
Chr. 22
E

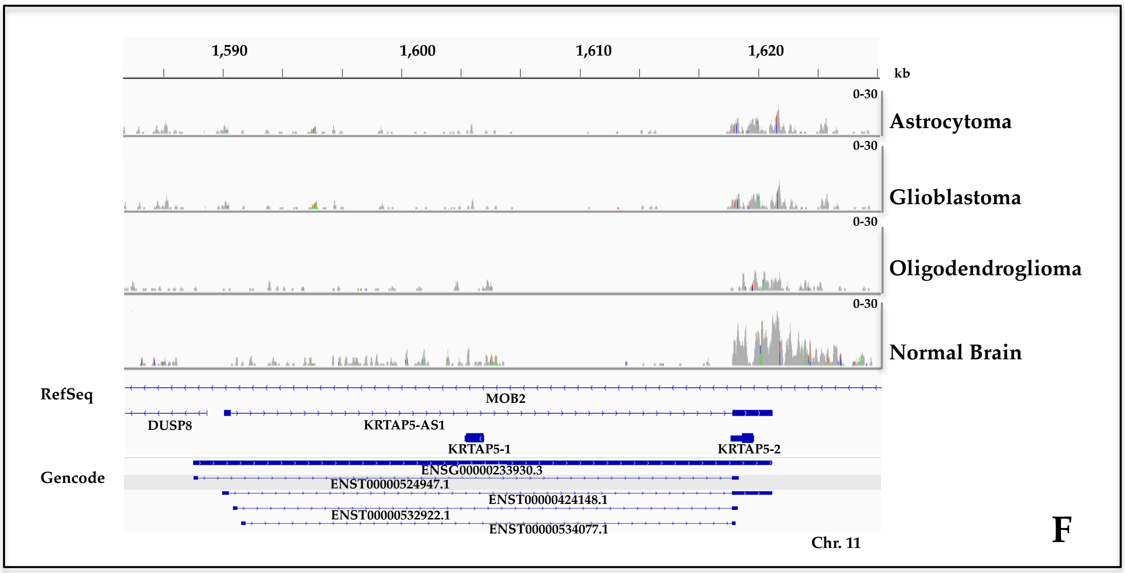
1,590
1,600
1,610
1,620
kb
0-30
Astrocytoma
0-30
Glioblastoma
0-30
Oligodendroglioma
0-30
Normal Brain
RefSeq
MOB2
DUSP8
KRTAP5-AS1
KRTAP5-1
KRTAP5-2
Gencode
ENSG00000233930.3
ENST00000524947.1
ENST00000424148.1
ENST00000532922.1
ENST00000534077.1
Chr. 11
F

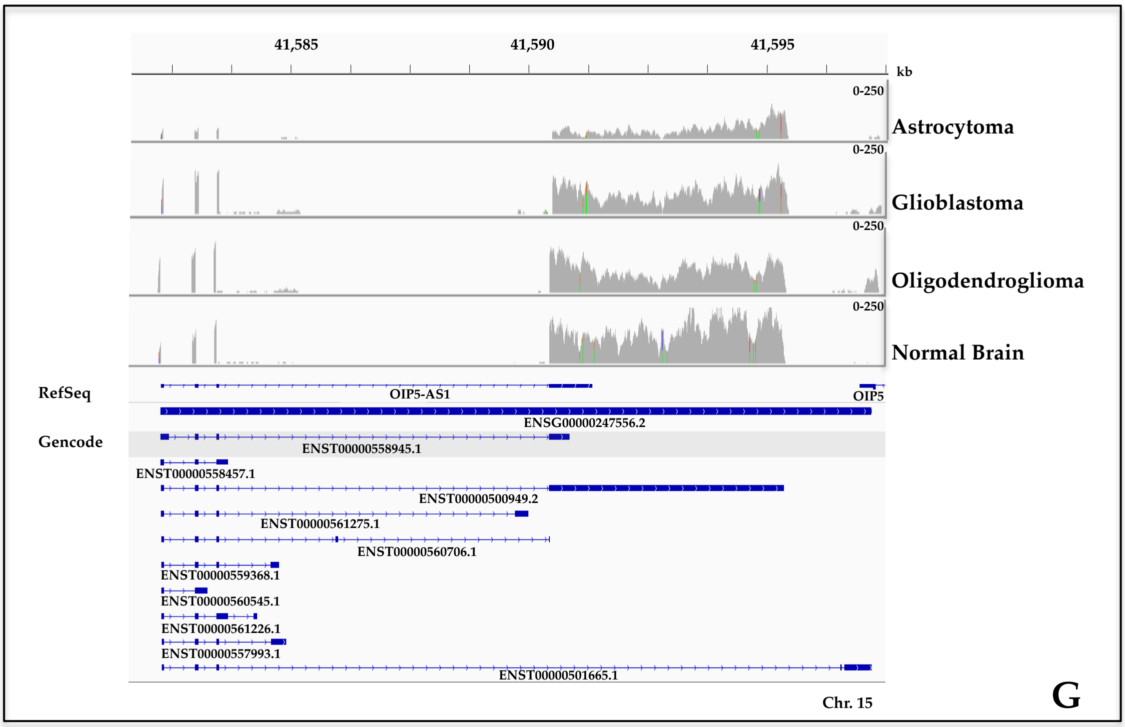
41,585
41,590
41,595
kb
0-250
Astrocytoma
Glioblastoma
Oligodendroglioma
Normal Brain
RefSeq
OIP5-AS1
OIP5
ENSG00000247556.2
Gencode
ENST00000558945.1
ENST00000558457.1
ENST00000500949.2
ENST00000561275.1
ENST00000560706.1
ENST00000559368.1
ENST00000560545.1
ENST00000561226.1
ENST00000557993.1
ENST00000501665.1
Chr. 15
G

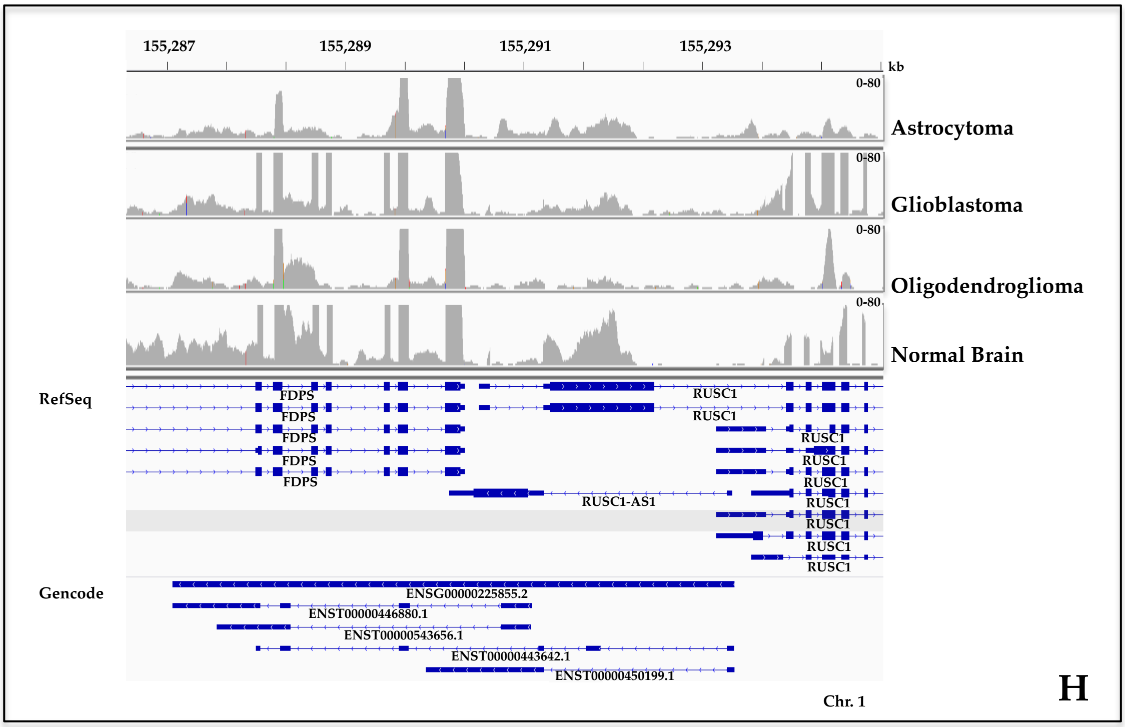
155,287
155,289
155,291
155,293
kb
0-80
Astrocytoma
Glioblastoma
Oligodendroglioma
Normal Brain
RefSeq
FDPS
RUSC1
RUSC1-AS1
Gencode
ENSG00000225855.2
ENST00000446880.1
ENST00000543656.1
ENST00000443642.1
ENST00000450199.1
Chr. 1
H

Figure S1. Genomic alignments of RNA-seq reads corresponding to the eight remaining lncRNAs in the three brain tumors types: (A) UHRF1, (B) UBL7-AS1, (C) DLEU2, (D) SYN2, (E) RFPL1S, (F) KRTAP5-AS1, (G) OIP5-AS1 and (H) RUSC1-AS1. In each panel the transcript annotations from both RefSeq and Gencode were displayed. The visualization of the alignments was obtained with the IGV software that did not operate any normalization of the read counts. For this reason it appears useful to remember the total number of reads aligned for each sample: 36.615.170 for Astrocytoma, 56.810.365 for Glioblastoma, 45.522.007 for Oligodendroglioma and 43.987.358 for Normal Brain.
