# Supplementary material for: An Association Rule Mining Approach to Discover lncRNAs Expression Patterns in Cancer Datasets
Source: Biomed Res Int. 2015 Jul 27;2015:146250. doi: 10.1155/2015/146250 (PMC4530207; doi:10.1155/2015/146250)
Supplement: Supplementary file 1 — Supplementary Material The list of comparisons from GEO datasets is provided in Table S1. The list of the 53 lncRNAs contained in at least one of the 102 nonredundant rules identified by the ARM analysis is provided in Table S2. The list of the 102 nonredundant rules identified through the ARM analysis is provided in Table S3. Number of comodulated genes for each of the 10 lncRNAs is provided in Table S4. Results of the CorrelaGenes analyses of the 10 lncRNAs are provided in Table S5. Results of the enrichment analyses of GO terms for the upregulated genes are provided in Table S6. Results of the enrichment analyses of GO terms for the downregulated genes are provided in Table S7. Figure S1. Genomic alignments of RNA-seq reads corresponding to the eight remaining lncRNAs in the three brain tumors types: (A) UHRF1, (B) UBL7-AS1, (C) DLEU2, (D) SYN2, (E) RFPL1S, (F) KRTAP5-AS1, (G) OIP5-AS1 and (H) RUSC1- AS1. In each panel the transcript annotations from both RefSeq and Gencode were displayed. Figure S2. Principal Component Analysis (PCA) performed on the GEO dataset GDS1962 (panels A and B) and ArrayExpress dataset E-GEOD-16011 (panels C and D) considering intensity values of all probes (panels A and C) or only probes corresponding to the 10 lncRNAs (panel B and D). Figure S3. Direct acyclic graph of the brain-specific Gene Ontology terms obtained by the analysis of downregulated genes. [file 146250.f1.zip › 146250.f1/Suppl_Fig2_revision.pdf]

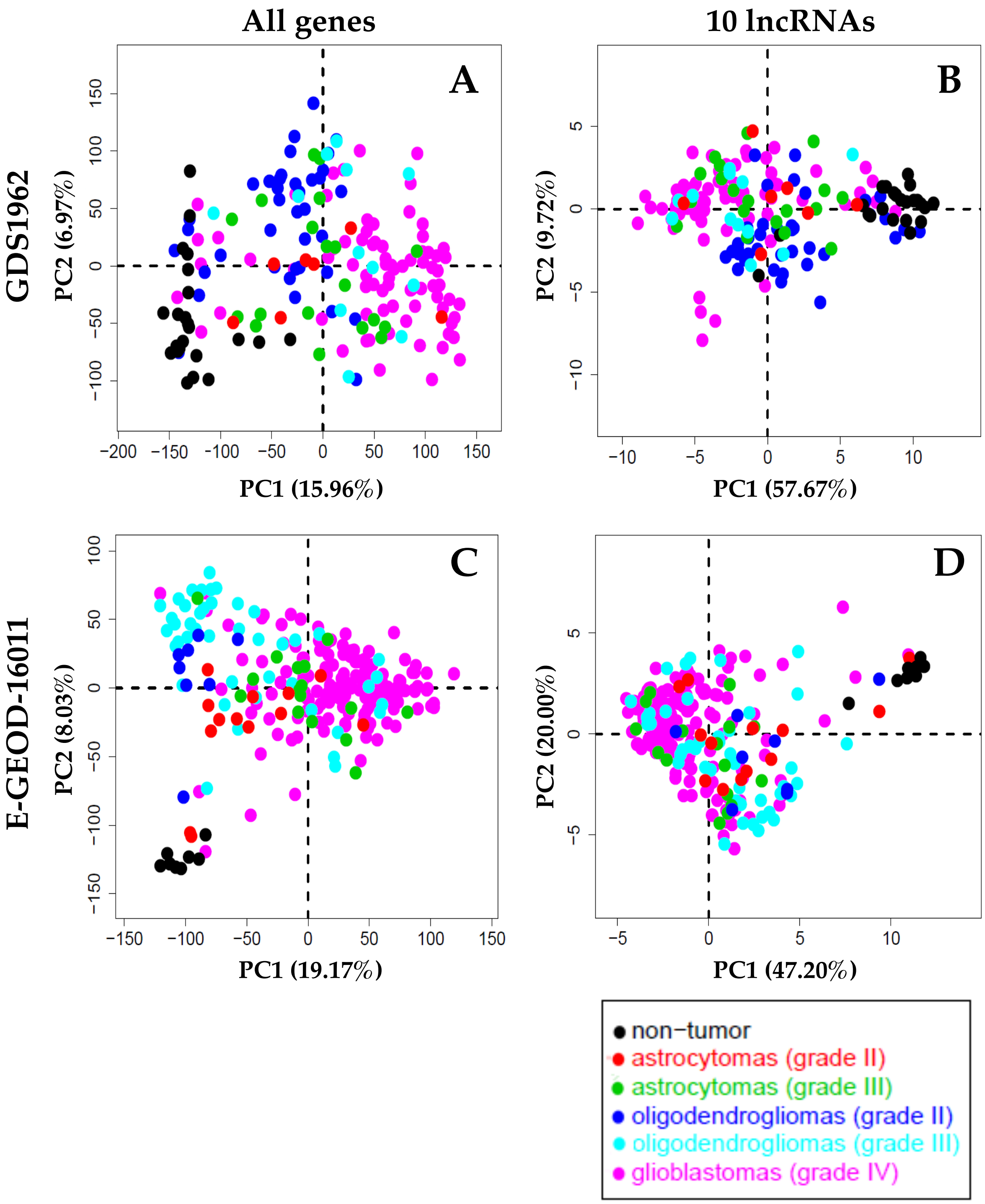

Figure S2. Principal Component Analysis (PCA) performed on the GEO dataset GDS1962 (panels A and B) and ArrayExpress dataset E-GEOD-16011 (panels C and D) considering intensity values of all probes (panels A and C) or only probes corresponding to the 10 lncRNAs (panel B and D).
